# Oral lichen planus after COVID-19, a case report

**DOI:** 10.1016/j.amsu.2021.103051

**Published:** 2021-11-11

**Authors:** Wafaa Saleh, Eman SHawky, Ghady Abdel Halim, Fatma Ata

**Affiliations:** Oral Medicine, Periodontology, Diagnosis and Oral Radiology Department, Faculty of Dentistry, Mansoura University, 33516, Egypt

**Keywords:** Lichen planus, COVID-19, Oral mucosa

## Abstract

**Introduction:**

The COVID-19 is a global pandemic that is now responsible for more than 3 million deaths around the world. The oral and dermatological manifestations of COVID-19 are being remarkably reported. This report aims to describe a unique case of oral presentation of erosive lichen planus after COVID-19 infection.

**Case presentation:**

We present a case of oral lichen planus manifested in the buccal mucosa and the tongue. The lesions were detected one month after COVID-19 infection. Clinical, as well as the histopathological diagnosis, was performed on the oral lesion to confirm oral lichen planus.

**Clinical discussion:**

Despite viruses being reported as a triggering factor in oral lichen planus, very few cases of oral lichen planus after COVID-19 infection were noticed. The immunological and psychological changes induced by COVID-19 infection may explain the tendency to find lichen planus lesions in COVID-19 patients.

**Conclusion:**

Our case suggests the possible association between COVID-19 and oral lichen planus. This report highlights the role of health practitioners to be concerned about the probably rising incidence of lichen planus during the COVID-19 pandemic and consider appropriate therapeutic and protective measures against infection.

## Highlights

• Coronavirus disease 2019 (Covid 19) causes pandemics all over the world.
• Oral and cutaneous manifestations of COVID-19 are emerging every day.
• Lichen planus is a new pattern of COVID‐19 associated lesion.
• Identification and diagnosis of lichen planus after COVID-19 need conducting entire clinical and laboratory investigation.

## 1 Introduction

Lichen planus is a chronic mucocutaneous inflammatory disease detected in 1–2% of the population. It affects mucous membranes and/or skin with a wide spectrum of clinical and histopathological manifestations. It is common in females more than males especially over 40 years old. The etiology of lichen planus is unknown, and its pathogenesis still needs further studies. It is known as an immune mediated disorder by T- T-lymphocytes [1,2].

The rising evidence shows that there are detected antigens on the surface of the basal cell layer of the epithelium that can trigger an abnormal cytotoxic T-cell reaction. Despite the limited data, these events have been related to exposure to contact allergens or infections as HCV [3].

Consequently, SARS-CoV-2 infection may represent an antigen that triggers this inflammatory cascade [4]. SARS-CoV-2 can induce wide alteration in the cellular as well as the humoral immune response [5].The immunological changes triggered by COVID-19 infection are similar to the alterations detected in the oral lichen planus. Consequently, in the long-term follows up of survivor patients of COVID-19 infection, oral health practitioners as well as dermatologists may detect an increased incidence of lichen planus [4].

Although wide varieties of oral manifestations have been detected after COVID-19 infection, very few cases of oral lichen planus were reported. In this report, we describe a unique case of oral erosive lichen planus that was detected in a male patient one month after COVID-19 infection.

### 1.1 Case presentation

A 63-year-old male patient was referred to the Oral Medicine and Periodontology Department, Faculty of Dentistry, Mansoura University, Egypt in April 2021. The patient was complaining of a burning sensation of the mouth especially the tongue and buccal mucosa.

The medical history was significant to chronic cholecystitis, right renal cyst, and right inguinal hernia. The patient had a COVID-19 infection one month before the oral lesion after traveling to another country. During COVID-19 infection, the patients reported various clinical signs and symptoms as sore throat, headache, hypogeusia, diarrhea, and bone ache.

He was advised to take his medications as well as staying at home until complete resolving the COVID-19 infection.

Intraoral examination revealed that there are painful erosive areas of the buccal mucosa and the dorsal surface of the tongue. The lesion of the left buccal mucosa shows a central erosive area surrounded by white radiating striae from the periphery of the lesion. (Fig. 1a, & 1b). The lesion is extremely painful and sensitive to touch resulting in difficulty eating and drinking.

**Fig. 1a:**
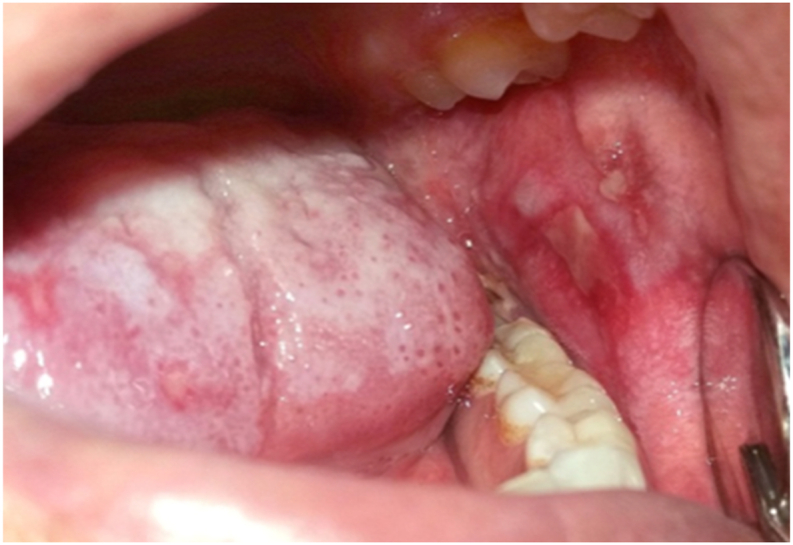
Clinical picture showing the oral erosive lichen planus lesion on the left side of buccal mucosa at baseline.

**Fig. 1b:**
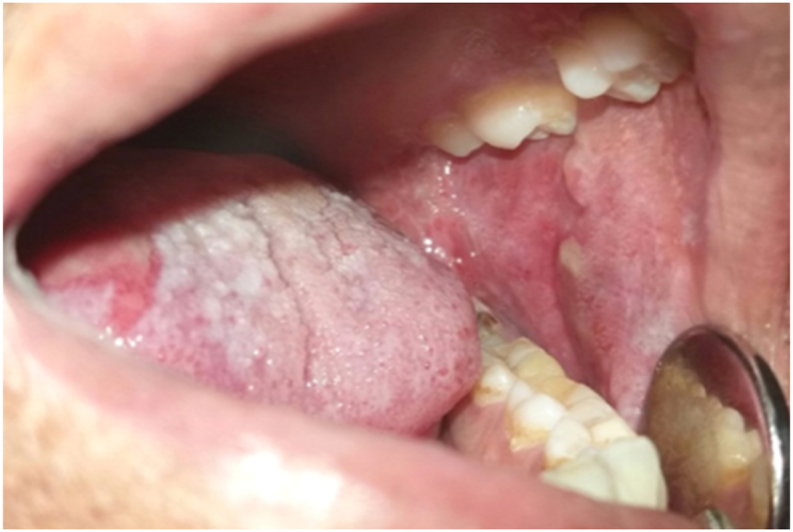
Clinical picture of the same case after 4 weeks of treatment showing marked healing of the erythematous and ulcerative area.

**Fig. 2:**
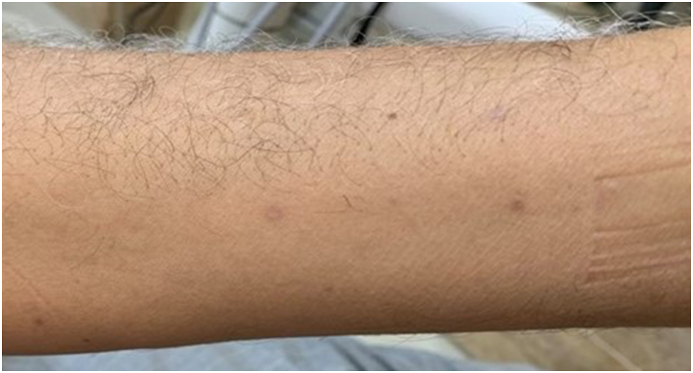
Clinical picture showing brown pruritic macules on the flexure surface of the arm of the same patient. (For interpretation of the references to colour in this figure legend, the reader is referred to the Web version of this article.)

Extraoral examination showed brown pruritic macules on the flexure surface of the arm that was detected with the oral lesion. Fig. 2 Painful palpable submandibular lymph nodes were detected while the other extraoral features were normal.

The patient denied the possible triggers of the lesions as medication and psychological stress, while he reported getting an infection of COVID-19 one month before the appearance of those lesions. Family history was negative for any systemic disease that might affect the occurrence of lichen planus.

**Fig. 3:**
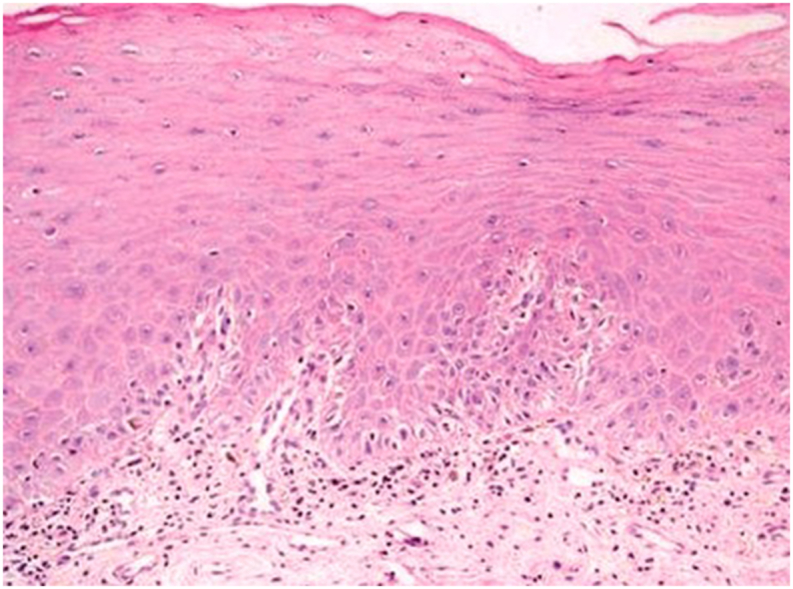
Photomicrograph of oral lichen planus showing inflammatory infiltrate forming a band in the superficial portion of the lamina propria (H&E x 200).

An incisional biopsy of the left buccal mucosa was obtained which revealed an oral lichen planus lesion with hyper-parakeratinized stratified epithelium, partially ulcerated, mild degree of acanthosis, liquefactive degeneration of basal cells, and prominent band like sub-epithelial lymphocytic infiltration which was compatible with the diagnosis of oral lichen planus. Fig. 3.

A skin biopsy was not performed according to the patient's desire. Additionally, similar lesions of cutaneous manifestations of lichen planus were reported in the literature [2]. Laboratory investigations showed normal WBCs count and high C-reactive protein. Serologic analyses were negative for hepatitis C virus. The patient was prescribed topical corticosteroids three times daily for 10 days after mealtime then gradual withdrawal of the medication was performed in response to the improvement and healing of the lesion. 4 weeks later, the patient showed marked improvement in the pain scores as well as a decrease in the size of the oral lesions. Fig. 1b.

For follow up of the case, the patient was asked to visit our clinic every two weeks for 2 months then one visit per month. This report is in line with the SCARE criteria [6].

## 2 Discussion

A wide variety of oral and skin manifestations have been detected in patients with a history of COVID-19 infection [4]. This report presents a unique case of oral lesions of erosive lichen planus which were detected one month after COVID-19 infection.

Various factors are affecting the oral mucosa resulting in appearance of different lesions. Stress, infections, and bad oral hygiene represent the most common factors disturbing the oral mucosa [7]. The surge in fear and stress during COVID-19 pandemic may be due to placing people in quarantine, travel restrictions, fear of losing livelihood and loved ones and phobia of contracting the infection [8].

Few reports of lichen planus among COVID-19 patients were reported. However, no report detected both oral and skin lesions of lichen planus in the same patient. In a retrospective study performed by Fidan et al., among 58 patients of COVID-19 who developed oral lesions, 12 patients developed oral lichen planus [9]. However, the authors didn't report that they performed a biopsy to confirm the diagnosis of oral lichen planus despite several diseases shows similar clinical manifestations to oral lichen planus. Interestingly, their study demonstrated that all patients had the disease in only one oral anatomic location. This is very uncommon for oral lichen planus as multiple locations are classically affected due to the multifocal nature of the disease [10]. Burgos-Blasco et al. reported a 56-year-old female with bilateral lace-like pattern of oral lichen planus on buccal mucosa without skin lesion after COVID-19 infection [4]. Diaz-Guimaraens et al. reported a case of 52 years old female with a pruritic solitary black annular skin lesion on the right shin that was noticed 5 days after the start of COVID-19 symptoms. In addition, a bilateral buccal mucosa showed reticular white lines in a lace-like pattern. A Skin Biopsy was performed to confirm the diagnosis of lichen Planus however the authors didn't report a biopsy from the oral lesion [11].

The immunological changes induced by COVID-19 infection may explain the tendency to find lichen planus lesions in COVID-19 patients [4]. The immunological alterations seen in lichen planus are similar to the changes triggered by COVID-19 virus. Consequently, a surge in the prevalence of lichen planus may be observed in the long-term survivors of COVID-19 [8,11].

COVID-19 was reported to cause immune dysregulation especially in the T- T lymphocyte subset resulting in reduced cellular immune response [5]. CD8T-cell in COVID-19 was found in a higher number and hyperactivated state as indicated by CD69, CD38 and CD44 expression [12,13].

Despite viruses were reported as a triggering factor in oral lichen planus, the association between oral lichen planus and COVID-19 may have been coincidental [14,15]. The hypothetical COVID-19 primed T-cells that could have cross-reacted with the epidermis-restricted antigens might have originated during the mild COVID- 19 episode and could have continued after that [11]. We recommend further studies including a large number of the population to be conducted to verify the association between COVID-19 and lichen planus.

## Study design

Case report.

## Ethical approval

The data presented in the current case report is reviewed and approved by the Ethical Committee at our center.

## Sources of funding

No sources of funding to declare.

## Authors contribution

**Wafaa Saleh:** Study concept, Data collection, Data analysis, Writing the paper. Revision.

**Eman SHawky:** Supervision and data validation, Revision.

**Ghady Abdel Halim:** Supervision and data validation, Revision.

**Fatma Ata:** Supervision and data validation, Revision.

## Patient's consent

Written informed consent was obtained from the patient for publication of this case report and accompanying images. A copy of the written consent is available for review by the Editor-in-Chief of this journal on request.

## Research registration

Not applicable**.**

## Guarantor

Wafaa Saleh is the Guarantor.

## Provenance and peer review

Not commissioned, externally peer-reviewed.

## Declaration of competing interest

The authors report no conflict of interest.

## References

[1] Mutafchieva M.Z., Draganova-Filipova M.N., Zagorchev P.I., Tomov G.T. (2018). Oral lichen planus - known and unknown: a review. Folia Med. (Plovdiv).

[2] Gorouhi F., Davari P., Fazel N. (2014). Cutaneous and mucosal lichen planus: a comprehensive review of clinical subtypes, risk factors, diagnosis, and prognosis. ScientificWorldJournal.

[3] Shiohara T., Mizukawa Y., Takahashi R., Kano Y. (2008). Pathomechanisms of lichen planus autoimmunity elicited by cross-reactive T cells. Curr. Dir. Autoimmun..

[4] Burgos-Blasco P., Fernandez-Nieto D., Selda-Enriquez G., Melian-Olivera A., De Perosanz-Lobo D., Dominguez-Santas M., Alonso-Castro L. (2021). COVID-19: a possible trigger for oral lichen planus?. Int. J. Dermatol..

[5] Qin C., Zhou L., Hu Z., Zhang S., Yang S., Tao Y., Xie C., Ma K., Shang K., Wang W., Tian D.S. (2020). Dysregulation of immune response in patients with coronavirus 2019 (COVID-19) in Wuhan, China. Clin. Infect. Dis..

[6] Agha R.A., Franchi T., Sohrabi C., Mathew G., Kerwan A. (2020). The SCARE 2020 guideline: updating consensus surgical CAse REport (SCARE) guidelines. Int. J. Surg..

[7] Martín Carreras-Presas C., Amaro Sánchez J., López-Sánchez A.F., Jané-Salas E., Somacarrera Pérez M.L. (2021). Oral vesiculobullous lesions associated with SARS-CoV-2 infection. Oral Dis..

[8] Routray S., Mishra P. (2020). A probable surge in oral lichen planus cases under the aura of coronavirus in females in India. Oral Oncol..

[9] Fidan V., Koyuncu H., Akin O. (2021). Oral lesions in Covid 19 positive patients. Am. J. Otolaryngol..

[10] Cheng Y.S., Gould A., Kurago Z., Fantasia J., Muller S. (2016). Diagnosis of oral lichen planus: a position paper of the American Academy of Oral and Maxillofacial Pathology. Oral Surg Oral Med Oral Pathol Oral Radiol.

[11] Diaz-Guimaraens B., Dominguez-Santas M., Suarez-Valle A., Fernandez-Nieto D., Jimenez-Cauhe J., Ballester A. (2021). Annular lichen planus associated with coronavirus SARS-CoV-2 disease (COVID-19). Int. J. Dermatol..

[12] Zheng H.Y., Zhang M., Yang C.X., Zhang N., Wang X.C., Yang X.P., Dong X.Q., Zheng Y.T. (2020). Elevated exhaustion levels and reduced functional diversity of T cells in peripheral blood may predict severe progression in COVID-19 patients. Cell. Mol. Immunol..

[13] Sun D., Li H., Lu X.X., Xiao H., Ren J., Zhang F.R., Liu Z.S. (2020). Clinical features of severe pediatric patients with coronavirus disease 2019 in Wuhan: a single center's observational study. World J Pediatr.

[14] Tenório J.R., de Camargo A.R., Lemos C., Ortega K.L. (2020). Oral lichen planus and HCV infection. Autops Case Rep.

[15] Lucchese A. (2015). A potential peptide pathway from viruses to oral lichen planus. J. Med. Virol..

